# Factors Associated With Mental Suffering in the Brazilian Population: A Multilevel Analysis

**DOI:** 10.3389/fpsyg.2021.625191

**Published:** 2021-03-25

**Authors:** Héllyda de Souza Bezerra, Roberta Machado Alves, Talita Araujo de Souza, Arthur de Almeida Medeiros, Isabelle Ribeiro Barbosa

**Affiliations:** ^1^Graduate Program in Public Health, Federal University of Rio Grande do Norte, Natal, Brazil; ^2^Graduate Program of Health Sciences, Federal University of Rio Grande do Norte, Natal, Brazil; ^3^Integrated Health Institute, Federal University of Mato Grosso do Sul, Campo Grande, Brazil

**Keywords:** psychological stress, mental health, socioeconomic factors, multilevel analysis, Brazil, mental suffering

## Abstract

**Purpose:** To analyze how individual characteristics and the social context are associated with mental distress symptoms in the Brazilian population.

**Method:** A multilevel cross-sectional study with data from the 2013 National Health Survey. There were two dependent variables: (a) decreased vital energy and somatic symptoms, (b) the presence of depressive thoughts. The independent variables were biological characteristics, education and income, habits and lifestyle, and context variables. Bivariate analysis was performed, and Prevalence Ratios calculated in a Poisson Regression (95% CI). A multilevel Poisson Regression was performed to verify the effect of individual and contextual variables.

**Results:** Regarding depressive thoughts, young and middle-aged individuals, low education, women, absence of partner, smokers or former smokers, and absence of health insurance were the categories at highest risk; belonging to classes D-E and living in states with lower expected years of schooling proved to be protective factors. Similar results were found for the second outcome.

**Conclusions:** Symptoms of mental distress were associated with the individual characteristics and contextual aspects of the federation unit. These findings indicate the importance of strengthening psychosocial care aimed at vulnerable groups.

## Introduction

Characterized by depressive symptoms, anxiety state, and a set of non-specific somatic complaints (Goldberg and Huxley, 1992), mental distress has been understood as a public health problem and object of discussion among health researchers due to alarming rates, for it has become the main source of disability adjusted life year (DALY) for women aged 15–24 (Senicato et al., 2018). In 2015, mental disorders accounted for 9.5% of the total DALY, occupying the 3rd and 1st position in the classification of DALY and years lived with disability (YLD) in the world, respectively, with emphasis on depressive and anxiety disorders (Bonadiman et al., 2017).

According to the World Health Organization (WHO), in the period from 2005 to 2015 there was an increase of 18.4% in the number of people with depression, and the prevalence of this health condition in Brazil is 5.8%, being the highest rate of Latin America. Regarding anxiety disorder, the worldwide prevalence is 3.6% and Brazil has the highest number of cases among all countries in the world, affecting 9.3% of the population (World Health Organization, 2017a). The highest prevalence of depression and anxiety are registered in the female population (Alves et al., 2015).

Common Mental Disorders (CMD) are characterized by depressive symptoms, states of anxiety, irritability, fatigue, insomnia, memory and concentration problems, and somatic complaints (Goldberg and Huxley, 1992). Results obtained in several national studies in the urban and rural context revealed a higher prevalence of CMD related to the women, low levels of education, low income, and separated, divorced, widowed or partner less women. Early and correct diagnosis of this disorder is essential to avoid physical and psychological damage to individuals and burden to the health system (Alves et al., 2015; Nunes et al., 2016).

Among the risk factors associated with the development of mental disorders, the abuse of alcohol and tobacco stands out, and in a survey conducted by the Ministry of Health of Brazil it was identified that 9.3% of the Brazilian population is a smoker and 13.7% do alcohol abuse (Garcia and Freitas, 2015; Parreira et al., 2017).

Factors commonly associated with mental disorders include not only individual attributes but also social, cultural, economic, political and environmental factors, and between 76 and 85% of people with mental disorders in low- and middle-income countries do not receive any kind of treatment (Goldberg and Huxley, 1992). The high number of low- and middle-income people who do not receive treatment can be justified because the individual needs to have access to the medication, and the lack of financial resources for its acquisition restricts or makes this access impossible, compromising the follow-up of the treatment of the mental disorder (Borba et al., 2018).

The negative consequences of mental disorders in the quality of life result from functional impairment with loss of work productivity and social isolation. They may lead to greater use of health services, which produces high costs for the health system and for individuals and their families, as well as less measurable costs such as individual and family suffering (Lima et al., 2008; Del'Olmo and Cervi, 2017).

The Psychosocial Care Network (RAPS), is part of the Unified Health System (SUS), is an articulated and effective network that assists different levels of health care, qualified to meet people in distress or presenting demands arising from mental disorders or chemical dependence. It works based on a territorial perspective, with emphasis on community-based services aimed at meeting the needs of users and their families, highlighting care centered on people's needs (Brasil, 2017).

In 2013, in order to monitor these themes, Brazil conducted the National Health Survey (NHS) with a national scope and at domestic level, which aims to produce data about the health situation and lifestyles of the Brazilian population in the country. The NHS was conducted in partnership with the Brazilian Institute of Geography and Statistics (IBGE) and the Ministry of Health of Brazil, and estimated that 7.6% of people aged 18 or older had been diagnosed with depression by mental health professionals, accounting for 11.2 million of people (Stopa et al., 2017).

Due to the numbers presented, discussing psychic suffering is relevant for understanding the reality and elaborating efficient action plans for mental health care of the affected demand. This research is relevant because of the need to discuss the theme to strengthen existing policies and build more effective measures that treat the right to mental health as a human right. In this sense, this study aimed to analyze the prevalence of mental distress symptoms in the Brazilian population and the association between individual characteristics and social context in a multilevel analysis.

## Materials and Methods

### Study Design and Database

This is an association study between dependent variables that express the presence of symptoms related to common mental distress and individual and contextual independent variables fitted in a Multilevel Poisson Regression model, where the first level corresponds to individual variables and the second to the contextual variables related to the Federation Unit.

This study used the National Health Survey (NHS) database of Brazil of the year 2013. This nationwide research was conducted to know and characterize through a household-based survey the Brazilian population profile in terms of the health situation, lifestyle, surveillance of chronic diseases, risk factors, access, and use of health services. The NHS uses an instrument designed specifically for the survey, consisting of three questionnaires: the household, the one related to all household residents, and the individual. All methodological details of the NHS are described elsewhere (Hanlon et al., 2008; van der Westhuizen et al., 2016).

### Characterization of Variables

#### Dependent Variable and Sample

The two dependent variables were built based on the set of symptoms related to Common Mental Disorders (CMD) and on the nomenclature of symptom classes described in the Self-Reporting Questionnaire (SRQ-20), already validated in several countries (Gonçalves et al., 2008; Santos et al., 2009; van der Westhuizen et al., 2016; Netsereab et al., 2018), including Brazil, and originally developed by Harding et al. (1980) to determine the physical and psycho-emotional symptoms related to CMD.

The SQR-20 has a cutoff point of 7/8 questions for the diagnosis of CMD (Gonçalves et al., 2008), and therefore for the construction of the dependent variables of this research, eight questions dealing with the perception of the health status of the Module N of NHS were selected. To elaborate the dependent variable called ‘Decreased vital energy and somatic symptoms’, the affirmative answers to at least four of the five selected questions (N10, N11, N13, N14, and N15) were considered. The second dependent variable, called “Depressive thoughts,” was built based on the affirmative answer to all the selected questions (N12, N16, and N17) (Table 1). In response, the individual had the option to report the absence of any of these symptoms.

**Table 1:** Description of the construction of the dependent variables according to the 2013 Brazilian National Health Survey.

**Variable**	**Id**.	**Question**
Decreased vital energy and somatic symptoms	N10	In the last 2 weeks, how often did you have sleep problems such as difficulty falling asleep, often waking up at night, or sleeping more than usual?
	N11	In the last two weeks, how often did you have problems not feeling rested and in good mood during the day, but rather tired and without energy?
	N15	How often did you feel slow to move or talk, or very agitated or restless in the last 2 weeks?
	N13	How did you have problems concentrating on your usual activities over the past 2 weeks?
	N14	How often did you have problems with your diet, such as lack of appetite or eating much more than usual in the last 2 weeks?
Depressive thoughts	N12	How often did you feel bothered about having little interest or not enjoying the things you do in the last 2 weeks?
	N16	How often did you feel depressed, down or hopeless in the last 2 weeks?
	N17	How often did you feel bad about yourself, finding yourself a failure or thinking that you have disappointed your family, in the last 2 weeks?

The decrease in vital energy and somatic symptoms represent physical symptoms and depressive thoughts represent psycho-emotional ones. Thus, the dependent variables were constructed with questions that were asked in the PNS and that were similar to those existing in the SQR-20.

Of the valid answers to the NHS questionnaire, 5,517 individuals answered positively to the questions described above that characterize the variable “decreased vital energy and somatic symptoms,” and 4,574 individuals answered positively to the characteristics of the variable “depressive thoughts.”

#### Independent Variables

##### Individual Level

The individual independent variables used in this study were: skin color; age group; gender; education level; marital status; practice of physical activity; smoking; alcohol use; have pets; have health insurance, and socioeconomic level according to the “Brazil Criteria for Economic Classification” of Brazilian Association of Research Companies (ABEP).

The “Brazil Criteria for Economic Classification” is based on the possession of goods, such as a car, computer, refrigerator, TV, washing machine, DVD player, microwave, motorcycle, and cell phone. There is also a bathroom, a maid, and the head of the family's educational level. Each item has different weights, and the variable represents the family's purchasing power. The score is defined between 0 and 100 points and divided into five classes (A, B, C, D, and E) in which class A represents the highest socioeconomic level while class E the lowest level.

This set of variables ensures the inclusion of individual characteristics, lifestyle, and socioeconomic conditions.

##### Contextual Level

For the contextual level, socioeconomic variables that can influence self-perceived health were selected. The variables included were Gini index, Human Development Index (HDI) and expected years of schooling for individuals over 18 years old related to the Federation Units.

**Figure 1:**
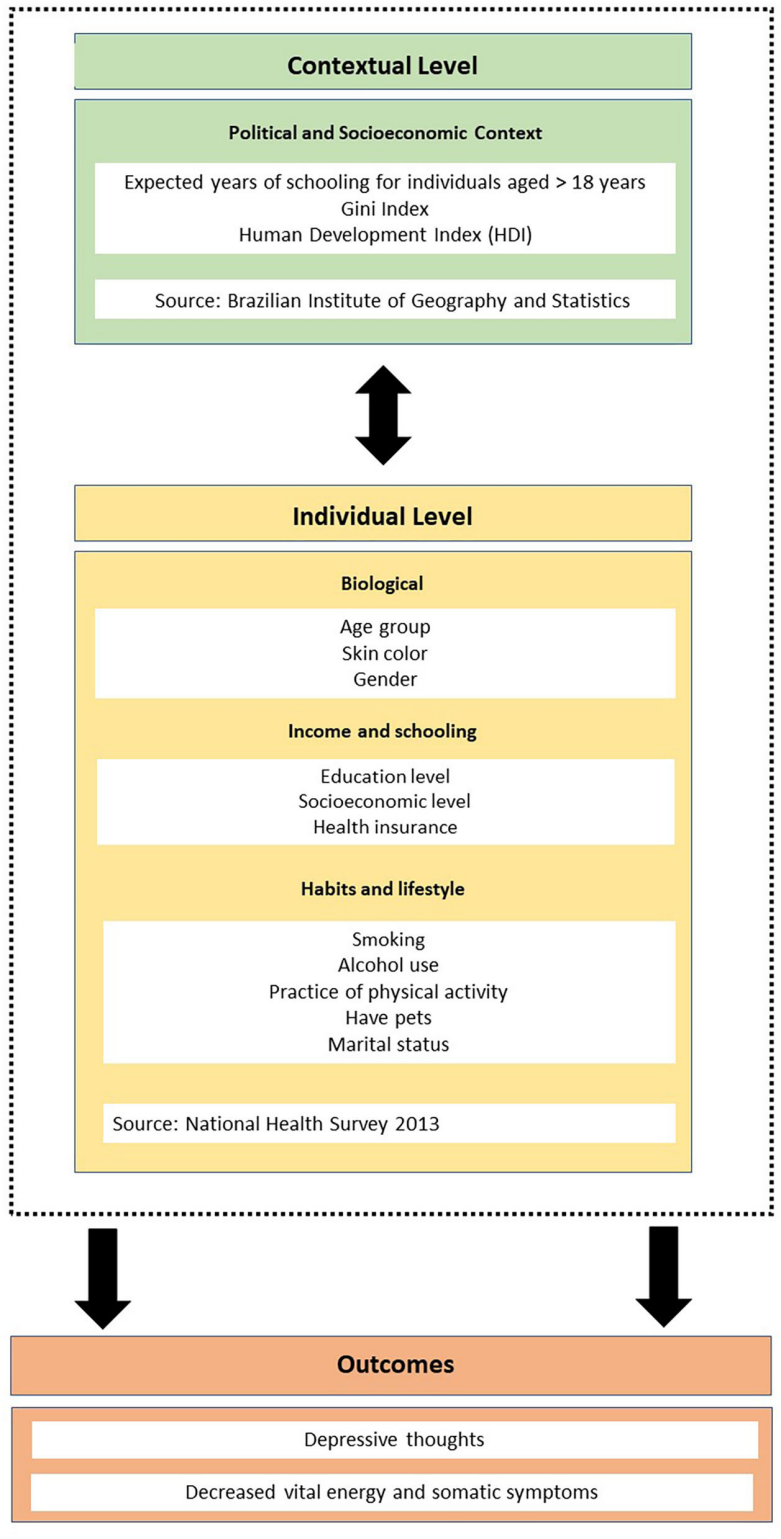
Framework of the study.

Figure 1 shows the relationship between outcomes and individual and contextual independent variables.

### Data Analysis

Prevalence rates were presented in absolute and relative values in each category, and the effect was estimated through Prevalence Ratios (PR) and their respective confidence intervals (95% CI). Variables with significant associations in bivariate analyses (*p* ≤ 0.20) were inserted in the multivariate model. In the first step, the contextual effect was evaluated based on a null model using the likelihood ratio test to verify significance. Two models were then tested, the first one with the individual level only, and the final model with the individual and contextual levels, with variables that remained significant in the model. Analyses were performed using the Stata 13 statistical software.

### Statement Ethics

The NHS was submitted to the National Research Ethics Committee of the National Health Council of the Ministry of Health of Brazil for approval and was approved under Protocol No. 328.159, in June 2013. At the end of the survey, all data were made available on the Ministry of Health website, becoming public domain.

Information related to contextual variables is also in the public domain and is available on federal government websites.

Thus, this study was developed based on a secondary database, which does not require the research ethics committee's appreciation, as provided by Resolution 510/2016 of the National Health Council (Brasil, 2016).

## Results

Table 2 shows the bivariate analysis results for the two outcomes studied and their relationship with individual and context variables. In “depressive thoughts,” no statistical significance was observed for the variables skin color and have pets. In “Decreased vital energy and somatic symptoms,” there was no significant association with the variable have pets. All other individual and contextual variables were included in the multivariate model to check the combined effect of individual and contextual co-variables.

**Table 2:** Bivariate association between outcomes and independent variables according to the 2013 Brazilian National Health Survey.

	**Depressive thoughts**			**Decreased vital energy and somatic symptoms**		
	***n*** **=** **4,574**			***n*** **=** **5,517**		
**Prevalence**	7.6% (95% CI 7.08–8.14)			9.16% (95% CI 8.46–9.91)		
**Variables**	***n*** **(%)**	***P***	**PR (95% CI)**	***n*** **(%)**	***p***	**PR**
**INDIVIDUAL LEVEL**						
Skin color						
White and others	1,917(7.65)	0.683	1	2,195 (8.76)	0.004	1
Black	2,657 (7.56)		1.05 (0.99–1.12)	3,322 (9.45)		1.09 (1.02–1.15)
Age group (years)						
18–24	515 (6.58)	<0.001	1	597 (7.63)	<0.001	1
25–39	1,532 (7.38)		1.11 (1.021–1.23)	1,861 (8.96)		1.17 (1.07–1.29)
40–59	1,765 (8.64)		1.3 (1.17–1.43)	2,009 (9.83)		1.29 (1.17–1.41)
60 and over	762 (6.82)		1.02 (0.91–1.14)	1,050 (9.39)		1.25 (1.11–1.36)
Gender						
Male	1,185 (4.57)	<0.001	1	1,528 (5.9)	<0.001	1
Female	3,389 (9.89)		2.16 (2.02–2.30)	3,989 (11.64)		1.97 (1.86–2.09)
Education level						
Illiterate	2,084 (8.65)	<0.001	1.60 (1.44–1.78)	2,602 (10.8)	<0.001	1.32 (1.20–1.44)
Primary school	736 (7.99)		1.49 (1.32–1.68)	748 (8.12)		1.00 (0.90–1.12)
Secondary school	1,326 (6.92)		1.28 (1.15–1.43)	1,535 (8.02)		0.99 (0.90–1.08)
Higher education	428 (5.52)		1	632 (8.15)		1
Marital status						
Live with partner	2.359 (6.83)	<0.001	1	2,937 (8.51)	<0.001	1
Does not live with partner	2,215 (8.63)		1.26 (1.19–1.34)	2,580 (10.05)		1.18 (1.12–1.24)
Practice physical activity						
Yes	1.161 (6.49)	<0.001	1	1,380 (7.71)	<0.001	1
No	3,413 (8.07)		1.26 (1.18–1.34)	4,137 (9.78)		1.27 (1.19–1.35)
Smoking						
Never smoked	2,772 (6.73)	<0.001	1	3,411 (8.28)	<0.001	1
Former smoker	934 (9.11)		1.34 (1.24–1.44)	1,138 (11.09)		1.33 (1.24–1.42)
Smoker	868 (9.94)		1.46 (1.35–1.58)	968 (11.09)		1.33 (1.24–1.43)
Alcohol use						
Yes	1,558 (6.77)	<0.001	0.82 (0.77–0.88)	1,832 (9.91)	<0.001	0.79 (0.75–0.84)
No	3,016 (8.11)		1	3,685 (7.96)		1
Have pets						
Yes	2,111 (7.69)	0.423	1	2,540 (9.26)	0.477	1
No	2,463 (7.52)		0.98 (0.92–1.04)	2,977 (9.09)		0.98 (0.93–1.04)
Health insurance						
Yes	1,000 (6.11)	<0.001	1	1,292 (7.89)	<0.001	1
No	3,574 (8.15)		1.37 (1.28–1.47)	4,225 (9.64)		1.20 (1.13–1.28)
Socioeconomic level						
Classes A-B	958 (7.35)	0.177	1	115 (8.55)	0.021	1
Class C	1,849 (7.84)		1.09 (1.01–1.18)	2,215 (9.39)		1.10 (1.02–1.18)
Classes D-E	1,767 (7.50)		1.05 (0.97–1.14)	2,187 (9.28)		1.07 (1.00–1.16)
**Contextual level**						
Gini index						
Up to 0.560	1,998 (7.76)	0.022	1	2,234 (8.67)	<0.001	1
From 0.560 to 0.610	1,808 (7.26)		0.94 (0.81–1.09)	2,332 (9.37)		1.06 (0.92–1.22)
From 0.620 and over	768 (8.04)		1.06 (0.87–1.28)	951 (9.96)		1.15 (0.95–1.38)
HDI						
0.735 and over	1,605 (8.08)	0.033	1	1,722 (8.66)	0.008	1
0.674–0.734	1,500 (7.55)		0.92 (0.77–1.09)	1,897 (9.55)		1.08 (0.91–1.27)
Up to 0.674	1,469 (7.18)		0.87 (0.73–1.03)	1,898 (9.28)		1.02 (0.86–1.21)
Expected years of schooling						
More than 9.4 year	2,149 (8.27)	<0.001	1	2,457 (9.45)	0.033	1
<9.4 years	2,425 (7.09)		0.84 (0.74–0.96)	3,060 (8.95)		0.91 (0.80–1.04)

*HDI, human development index*.

In Tables 3, 4, it is possible to observe that there was a contextual effect at the federation unit level for both outcomes, since the likelihood ratio was significant for both, showing the adequacy of the sample for this analysis.

**Table 3:** Multilevel poisson regression analysis for the outcome “Depressive thoughts” in the 2013 Brazilian National Health Survey.

	**Null model (*n* = 4,574)**	**Model 1 (*n* = 4,574)**	**Final model (*n* = 4,574)**			
	**PR (95% CI)**	***p***	**PR (95% CI)**	***p***	**PR (95% CI)**	***p***
**INDIVIDUAL LEVEL**						
Age groups (years)						
25–39			1.15 (1.04–1.27)	0.006	1.15 (1.04–1.28)	0.005
40–59			1.20 (1.085–1.33)	<0.001	1.21 (1.09–1.34)	<0.001
60 and over			0.84 (0.74–0.94)	0.005	0.85 (0.75–0.96)	0.009
Women			2.26 (2.11–2.43)	<0.001	2.29 (2.15–2.45)	<0.001
Education level						
Illiterate			1.50 (1.33–1.69)	<0.001	1.52 (1.35–1.71)	<0.001
Primary school			1.41 (1.24–1.61)	<0.001	1.43 (1.26–1.62)	<0.001
Secondary school			1.24 (1.11–1.39)	<0.001	1.25 (1.11–1.40)	<0.001
Does not live with partner			1.25 (1.17–1.33)	<0.001	1.24 (1.17–1.32)	<0.001
Smoking						
Former smoker			1.47 (1.36–1.59)	<0.001	1.46 (1.35–1.58)	<0.001
Smoker			1.54 (1.42–1.68)	<0.001	1.53 (1.41–1.65)	<0.001
Socioeconomic level						
Class C			0.98 (0.90–1.06)	0.713	0.98 (0.90–1.07)	0.757
Classes D-E			0.90 (0.82–0.98)	0.022	0.90 (0.83–0.98)	0.027
Does not have health insurance			1.17 (1.08–1.27)	<0.001	1.18 (1.09–1.28)	<0.001
Does not perform physical activity			1.03 (0.96–1.10)	0.383		
Uses alcohol			0.95 (0.89–1.02)	0.220		
**CONTEXTUAL LEVEL**						
Expected years of schooling						
More than 9.4 year					1	0.007
<9.4 years					0.84 (0.74–0.95)	
**Random effects**	**Variance (95% CI)**		**Variance (95% CI)**		**Variance (95% CI)**	
Department level	0.028	0.014–0.055	0.027	0.014–0.054	0.020	0.009–0.041
LR test (χ2; *p*)	65.56	*p* < 0.001	62.11	*p* < 0.001	39.56	*p* < 0.001

**Table 4:** Multilevel poisson regression analysis for the outcome “Decreased vital energy and somatic symptoms” in the Brazilian National Health Survey 2013.

	**Null model (*n* = 5,517)**	**Model 1 (*n* = 5,517)**	**Final Model (*n* = 5,517)**			
	**PR (95% CI)**	***p***	**PR (95% CI)**	***p***	**PR (95% CI)**	***p***
**INDIVIDUAL LEVEL**						
Age groups (years)						
25–39			1.14 (1.04–1.25)	0.005	1.14 (1.04–1.25)	0.005
40–59			1.12 (1.02–1.23)	0.017	1.12 (1.02–1.23)	0.016
60 and over			0.95 (0.85–1.06)	0.367	0.95 (0.85–1.06)	0.382
Women			2.02 (1.90–2.15)	<0.001	2.02 (1.90–2.15)	<0.001
Education level						
Illiterate			1.21 (1.09–1.33)	<0.001	1.21 (1.09–1.33)	<0.001
Primary school			0.96 (0.86–1.07)	0.528	0.96 (0.86–1.08)	0.538
Secondary school			0.96 (0.87–1.06)	0.492	0.96 (0.87–1.06)	0.497
Does not live with partner			1.15 (1.09–1.22)	<0.001	1.15 (1.09–1.22)	<0.001
Does not perform physical activity			1.07 (1.00–1.14)	0.025	1.07 (1.00–1.14)	0.025
Smoking						
Former smoker			1.42 (1.32–1.54)	<0.001	1.42 (1.32–1.54)	<0.001
Smoker			1.43 (1.32–1.52)	<0.001	1.43 (1.32–1.52)	<0.001
Uses alcohol			0.92 (0.87–0.98)	0.015	0.92 (0.87–0.98)	0.016
Does not have health insurance			1.07 (1.00–1.15)	0.034	1.07 (1.00–1.15)	0.040
**CONTEXTUAL LEVEL**						
Gini index						
From 0.520 to 0.610					1.03 (0.90–1.18)	0.632
From 0.620 and over					1.11 (0.93–1.33)	0.021
**Random effects**	**Variance (95%CI)**		**Variance (95%CI)**		**Variance (95%CI)**	
Department level	0.026	0.013–0.049	0.023	0.012–0.044	0.021	0.011–0.041
LR test (χ^2^; *p*-value)	98.19	*p* < 0.001	81.51	*p* < 0.001	74.47	*p* < 0.001

For the outcome ‘depressive thoughts’ (Table 3), model 1 with individual variables revealed that young, middle-aged, low-educated female adults who did not live with a partner, who were smokers or former smokers, and those who did not have health insurance were the categories at highest risk and remained significant in the model 1. With the inclusion of the variable “expected years of schooling” in the final model, all the variables described above remained significant in the model, with emphasis on the characteristics of belonging to income classes D and E and residing in states that have lower expected years of schooling, both representing protective factors. Human development index and Gini Index lost statistical significance and were excluded from the final model. The observed variation for the model that included the state level ranged from 0.027 to 0.020, indicating an important effect between states.

As for the outcome “Decrease vital energy and somatic symptoms” presented in Table 4, the results were quite similar. Unlike the previous model, in this outcome, the variables “alcohol use” and “not performing physical exercise” were inserted in the model 1 and remained significant in the final model, the first as a protective factor and the second as a risk factor. Regarding the contextual effect, the Gini index was inserted in the final model, but lost statistical significance.

## Discussion

The multilevel analysis conducted in this study showed that most of the individual characteristics studied were directly linked to depressive thoughts and decreased vital energy. Young and middle-aged adults, women, low-educated individuals, individuals who do not live with a partner, smokers, and former smokers, and individuals who did not have health insurance were associated with depressive thinking symptoms and decreased vital energy, according to the present study.

No association was observed between mental distress and skin color. However, the literature shows a direct association between race or skin color and mental health, and these differences are related to the disadvantages faced by black people in Brazil, which often turn into risk factors for mental illnesses (Smolen and Araújo, 2017). The study by Madeira and Gomes (2018) shows that the black race is a vulnerable population due to its socioeconomic and cultural context resulting from the process of centuries of slavery in Brazil. Black people have mostly lower education, lower positions in the labor market, and less access to health services. These aspects lead to a greater propensity to get sick; thus, this population deserves to be treated with greater importance in public health policies.

The present study showed that depressive thoughts and decreased vital energy and somatic symptoms were not associated with the variable “have pets.” Regarding the characteristic of living with pets, the literature shows that animals, especially dogs, can contribute directly to the improvement and conviviality of people with mental illnesses; however, these animals need to be trained by specific organizations to effectively provide such assistance. However, little is known about the effect of this contribution in the long term (Lloyd et al., 2019).

A population-based study conducted in Brazil showed a high prevalence of symptoms of depression and mood disorders in the middle-aged Brazilian population (Barros et al., 2017), as well as the data found in the NHS conducted in Brazil in 2013, which was used in the present research. Depressive symptoms in the middle-aged population are a public health problem in many countries of the Americas, with emphasis on the United States and the entire Latin American region (An and Xiang, 2015).

As for gender, depression is more prevalent in women, and this is a worldwide fact. Data from the WHO show that on average 5.1% of women and 3.6% of men are affected by this disorder (World Health Organization, 2017b). The NHS conducted in Brazil in 2013 showed the high prevalence of depressive symptoms in Brazilian women, especially those living in urban areas, with low levels of education, who had chronic diseases, and who had difficulty in accessing mental health care (Lopes et al., 2016). Another study conducted in Brazil also confirms the data indicated by the present research on the higher prevalence of depressive disorders in adult women, people who do not have a partner, and users of cigarettes and alcohol (Gonçalves et al., 2018). The literature explains that the prevalence of symptoms of depressive disorders are higher in women due to physiological and hormonal differences and factors such as low educational level, low income and socio-cultural issues (Silva et al., 2014; Stopa et al., 2015).

As previously mentioned, low education is a factor associated with mental distress and consequently depressive thoughts. Individuals with depressive disorders and thoughts tend to have less education, thus lower social and economic status, and they are more vulnerable to a worse perception of their health status and quality of life (Cho et al., 2019). In the health context, socioeconomic status is also an important factor for access to private assistance. According to the 2013 NHS, most people who have a private plan in Brazil have good economic conditions, complete higher education, live in urban areas, and have a good self-reported health condition (Malta et al., 2017a,b). Thus, the association between depressive thoughts and absence of private health plan is understandable, because the absence of supplementary health may justify a greater difficulty in accessing services and consequently greater incidence of illnesses.

As for individuals who do not live with a partner, loneliness and lack of contact with people has been an object of concern and an unfavorable behavior to mental health, especially in middle-aged adults (Hämmig, 2019). In the last decades, there has been a significant increase in the number of people who live alone, who are isolated, and have no partners. These lonely people are vulnerable to social isolation and are likely to require psychological care due to depressive feelings (Klinenberg, 2016).

As for cigarette use, this aspect has a strong association with depressive symptoms. Individuals who smoke have a high prevalence of diagnoses of minor depression and major depression, as well as depressive mood lasting longer than seven days (An and Xiang, 2015). However, the literature also indicates that quitting smoking may lead the individual to develop depressive symptoms and thoughts (van der Meer et al., 2013). These findings may explain the association found in the study that both smokers and former smokers may be affected by depressive thoughts and decreased vital energy.

In addition to risk factors, the present study also showed characteristics that as acted as protective factors for the outcome variables, such as belonging to lower income classes and residing in states that have lower expected schooling for the variable “depressive thoughts.” In contrast, population-based studies conducted in Brazil have shown that income, low educational attainment, and the onset of depressive thoughts are directly linked; being in a low economic condition can often determine the onset of these symptoms because factors such as education and poor access to health services are risk factors for acquiring illnesses (Stopa et al., 2015; van der Westhuizen et al., 2016; Malta et al., 2017a).

It is assumed that the findings of the present research may be influenced by other variables such as religion, family context and social interaction in the community. Societies facing difficult living conditions often have greater religiousness and greater social iteration, demonstrating high levels of well-being and happiness, which may act as a protective factor for the development of mental disorders (da Silva and dos Santos, 2013).

Regarding the variable “decreased vital energy and somatic symptoms,” its characteristic ‘alcohol use’ acted as a protective factor. It is noteworthy that the association between alcohol and mood disorders may vary over time and between different ages, showing a distinct relationship in adolescents and adults, besides differences between sexes. It should also be considered that people with mental disorders may have changed their habits and reduced their alcohol consumption. However, in short, alcohol use can predispose the population at risk of developing mood problems and depressive symptoms by causing decreased vital energy (Pedrelli et al., 2016).

Physical inactivity was considered a risk factor for depressive symptoms and consequently decreased vital energy. The prevalence of mental disorders is higher in individuals who did not practice physical activity, as such activity promotes the improvement of well-being and mental health (Lourenço et al., 2017).

Regarding the contextual factors, the expected years of schooling, the Human Development Index (HDI) and the Gini index were not associated with the studied outcomes. It was thus demonstrated that these context variables have little explanatory power over the prevalence of mental suffering in the Brazilian population.

One of the limitations of this study has to do with the response bias, since the theme is surrounded by ethical, moral and cultural issues, and these factors strongly influence the responses of people. There may have been omission of information on mental distress when individuals were questioned directly, out of fear or embarrassment of exposure. However, population surveys provide us with representative samples of the population from all regions of the country and can serve to compare these results with those of other countries, as well as support the organization of public policies aimed at minimizing the problem. The prevention of these events represents a major challenge because of the need for the response to articulate different areas, requiring interdisciplinary action, as well as the involvement of various sectors of civil society and governmental organizations.

Recognizing the magnitude of common mental disorders in Brazil through population-based researches and a methodology that takes into account each individual's context is invaluable for assessment and planning of the health system, contributing to the advancement of knowledge and improvements in health care policies.

The present study reveals that symptoms of mental distress were mainly associated with individual characteristics such as young and middle-aged adults, women, low education, absence of partner, smoking habit, and absence of health insurance. Belonging to income classes D and E and living in states with lower expected years of schooling proved to be protective factors for the outcome variable “depressive thoughts.” Context variables lost their significance after the final multilevel analysis model.

The present findings indicate the importance of strengthening the health care network, especially in psychosocial care services aimed at the most vulnerable groups, and thus develop preventive strategies for these groups in order to reduce the incidence rates of mental illnesses. Therefore, the present research is of fundamental importance for the area of collective public health because it stimulates a reflection on mental health policies in Brazil, influencing their re-evaluation and restructuring. It is necessary that further studies be developed on this theme in Brazil and in other countries in order to seek innovations and other relevant findings in this area.

## Data Availability Statement

The original contributions presented in the study are included in the article, further inquiries can be directed to the corresponding authors.

## Author Contributions

HSB and RMA contributed to the conceptualization, data curation, formal analysis, and methodology. IRB contributed to the conceptualization, data curation, formal analysis, methodology, supervision, and validation. TAS and AAM contributed to the data curation, formal analysis, and methodology. All authors contributed to the writing and final approval of the manuscript.

## Conflict of Interest

The authors declare that the research was conducted in the absence of any commercial or financial relationships that could be construed as a potential conflict of interest.
